# Tackling a difficult question: how do crystals of coordination polymers form?

**DOI:** 10.1107/S2052252514018624

**Published:** 2014-08-29

**Authors:** Stuart L. James

**Affiliations:** a School of Chemistry and Chemical Engineering, Queen’s University Belfast, David Keir Building, 39 Stranmillis Road, Belfast BT9 5AG, UK

**Keywords:** crystallization, ring-opening polymerization, coordination polymers

## Abstract

Some questions are hard to address, and equally hard to ignore. Recent work by C. Y. Su *et al.* [Jiang *et al.* (2014), *IUCrJ*, 305–317] concerns a highly challenging aspect of crystalline coordination polymers – trying to understand how they form.

Some questions are hard to address, and equally hard to ignore. Recent work by C. Y. Su *et al.* (Jiang *et al.*, 2014 ▶) concerns a highly challenging aspect of crystalline coordination polymers – trying to understand how they form. Coordination polymers (also known as metal organic frameworks, or MOFs) are extended structures based on alternating metal ions and organic bridging ligands. The advent of these materials has been one of the most important developments in chemistry over recent years. More than 4000 papers were published about them last year alone.^1^ In terms of applications, a critical point is that some of these materials are highly porous, exhibiting record-breaking internal surface areas and gas-storage capacities. They are now produced commercially and industrial applications are anticipated over the next few years. However, virtually all research in the area concerns their structures and their properties. Although structural work requires high expertise and presents challenges, it is feasible, especially if the materials are crystalline. We have the technology (X-ray diffractometers being powerful tools in this regard).

But what about how these extended structures actually form? This is far harder to address. For this, we do not have the technology (which tools might enable the visual­ization of such crystal growth at a molecular level?). The work reported by Su *et al.* tries to shed greater light on this difficult question.

To go back a step in the story, something over ten years ago, the groups of Richard Puddephatt (Brandys & Puddephatt, 2001 ▶) and myself (Lozano *et al.*, 2001 ▶) independently noted that the solutions from which crystals of coordination polymers grow sometimes contain well defined coordination rings, or cages, rather than polymers. It was further intriguing that the polymers which crystallized from these solutions could be structurally related to their discrete precursors by ring-opening polymerization (ROP) processes. ROP is very well known in other areas of chemistry. An age-old example is the transformation of S_8_ rings into long sulfur chains which occurs on heating. ROP is also used to manufacture organic polymers – Chauvin, Grubbs and Schrock were awarded the 2005 Nobel Prize in Chemistry for work which included catalytic ring-opening polymerization of cyclic alkenes (Chauvin *et al.*, 2005 ▶). Manners *et al.* have also pioneered the ring-opening polymerization of strained metallocenes to give metal organic polymers (Foucher *et al.*, 1992 ▶). However, it seemed at that time that ROP had not yet been put forward as a way in which crystalline coordination polymers could form.

How can evidence be obtained to support or disprove this possible mechanism? Where might such polymerization occur – in solution, followed by crystallization, or potentially at the surface of the crystal itself…?

The paper by Su *et al.* describes some careful observations of such a ‘coordination ROP’ system. Their chemistry is based on silver ions and an organic molecule (ligand) which can bridge between the silver ions. Their system is flexible enough to give either rings or chains (polymers) depending on the conditions.

In essence Su *et al.* have discovered at least two remarkable and interesting things about the crystals they obtained in their work. Firstly, they found that the crystals consisted not purely of the cyclic or the polymeric forms of this complex, but both forms together – the crystals obtained are an unusual sort of solid solution (see Fig. 1 ▶). The relative amounts of the ring and polymeric forms varied depending on the crystallization conditions. This is intriguing to think about. One possibility which it suggests is that crystallization may have occurred such that discrete cycles underwent ROP as they collided with the surface of the growing crystal (to give the polymeric component) but that simultaneously some cyclic complexes collided but did not ring-open and so became incorporated (trapped) into the growing crystal unchanged. The fact that stable solid solutions could form based on these two seemingly quite different components is due to the serendipitously close similarity between the cyclic structure and pairs of monomers in adjacent chains.

The second interesting observation was that crystals containing the cyclic form underwent a phase change upon heating. Although the structure of the high-temperature phase could not be determined, it is possible that this phase change involved a solid-state ring-opening polymerization. If so, this would suggest another possible mechanism by which crystals of coordination polymers form. Potentially, under prolonged standing during crystallization from solution, crystals of discrete structures could form first, but then spontaneously polymerize by ROP within the bulk of the crystal. These are possibilities. It needs to be noted, that in reality, partly because of the great variety of metal ions and organic bridging ligands which can form coordination polymers, there is unlikely to be one single mechanism that will describe the formation of all such materials.

The work by Su *et al.* stands out since it addresses a challenging and fundamental aspect of this area of materials science – no less than ‘how do these crystals form?’. Furthermore, it does actually provide us with further tantalizing clues about those fleeting events which may occur during the formation of crystals from solution. One day we may have the tools to visualize such processes at the molecular level with comparative ease. Until then, we must infer what we can from what we can see before and after crystallization has happened – the rewards of original insights are still there – think hard!

## Figures and Tables

**Figure 1:**
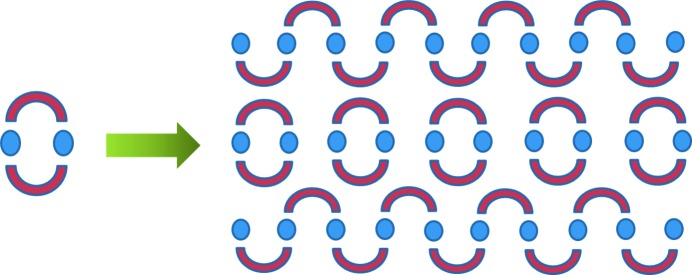
Transformation of rings into polymers on crystallization from solution; Su *et al.* observed that sometimes rings can cocrystallize together with polymers.

## Footnotes

1 From a Web of Science search for 2013 for papers with ‘metal organic framework’ or ‘metal organic frameworks’ or ‘coordination polymer’ or ‘coordination polymers’ as the search term.
